# CTIP2 Expression in Human Head and Neck Squamous Cell Carcinoma Is Linked to Poorly Differentiated Tumor Status

**DOI:** 10.1371/journal.pone.0005367

**Published:** 2009-04-28

**Authors:** Gitali Ganguli-Indra, Christine Wasylyk, Xiaobo Liang, Regine Millon, Mark Leid, Bohdan Wasylyk, Joseph Abecassis, Arup Indra

**Affiliations:** 1 Department of Pharmaceutical Sciences, College of Pharmacy, Oregon State University, Corvallis, Oregon, United States of America; 2 Centre Paul Strauss, Strasbourg, France; 3 Environmental Health Sciences Center, Oregon State University, Corvallis, Oregon, United States of America; 4 IGBMC, Inserm U596 and CNRS UMR 7104, Illkirch, France; 5 Université Louis Pasteur, Strasbourg, France; Karolinska Institutet, Sweden

## Abstract

**Background:**

We have demonstrated earlier that CTIP2 is highly expressed in mouse skin during embryogenesis and in adulthood. CTIP2 mutant mice die at birth with epidermal differentiation defects and a compromised epidermal permeability barrier suggesting its role in skin development and/or homeostasis. CTIP2 has also been suggested to function as tumor suppressor in cells, and several reports have described a link between chromosomal rearrangements of CTIP2 and human T cell acute lymphoblast leukemia (T-ALL). The aim of the present study was to look into the pattern of CTIP2 expression in Head and Neck Squamous Cell Carcinoma (HNSCC).

**Methodology/Principal Findings:**

In the present study, we analyzed CTIP2 expression in human HNSCC cell lines by western blotting, in paraffin embedded archival specimens by immunohistochemistry (IHC), and in cDNA samples of human HNSCC by qRT-PCR. Elevated levels of CTIP2 protein was detected in several HNSCC cell lines. CTIP2 staining was mainly detected in the basal layer of the head and neck normal epithelium. CTIP2 expression was found to be significantly elevated in HNSCC (p<0.01), and increase in CTIP2 expression was associated with poorly differentiated tumor status. Nuclear co-localization of CTIP2 protein and cancer stem cell (CSC) marker BMI1 was observed in most, if not all of the cells expressing BMI1 in moderately and poorly differentiated tumors.

**Conclusions/Significance:**

We report for the first time expression of transcriptional regulator CTIP2 in normal human head and neck epithelia. A statistically significant increase in the expression of CTIP2 was detected in the poorly differentiated samples of the human head and neck tumors. Actual CTIP2, rather than the long form of CTIP2 (CTIP2_L_) was found to be more relevant to the differentiation state of the tumors. Results demonstrated existence of distinct subsets of cancer cells, which express CTIP2 and underscores the use of CTIP2 and BMI1 co-labeling to distinguish tumor initiating cells or cancer stem cells (CSCs) from surrounding cancer cells.

## Introduction

Head and neck squamous cell cancers (HNSCC) are the sixth most common cancers in the world and are a major cause of significant morbidity. In the United States alone, Head and neck cancers account for less than 5% of all cancers and for less than 3% of all cancer deaths. For cancers of the oral cavity and pharynx alone 35,310 new cases and 7,590 deaths are estimated for 2008 [1], [2]. Although, HNSCC has been linked to tobacco and betel nut use, alcohol consumption, frequent mouthwash use, exposure to human papillomavirus (HPV), and people with history of smoking and alcohol use are at a greater risk [2], [3]. Genetics and other risk factors may also be associated with the pathogenesis of the disease. New approaches in the treatment of locally advanced HNSCC include chemotherapy and curative treatment to achieve organ preservation and to improve overall survival [4]. Inspite of advances in surgical and other treatments that enhance quality of life and moderating pain, survival rates are not improving for this type of cancer [5], [6]. There has been no significant improvement in 5-year survival over the past 20 years, despite aggressive and multidisciplinary treatment approaches [7], [8], [9]. Research on Molecular biology has provided us with large number of biomarkers that can provide information related to prognosis, treatment options, recurrence and development of secondary primary tumor [10]. Although biomarkers like human papillomavirus (HPV) and EGFR are well studied, their use in therapeutics is still not exploited successfully for efficient management of head and neck cancer [10]. New techniques are being developed to analyze simultaneously huge number of markers which should expand our knowledge of the biology of these cancers. Basic research efforts in terms of identifying novel genes with an altered expression pattern and having a role in critical biological processes such as proliferation and differentiation, disease progression and metastasis of tumors are needed to facilitate the diagnosis, prognosis and therapy of HNSCC patients.

Chicken ovalbumin upstream promoter-transcription factor (COUP-TF)-interacting protein 2 (CTIP2, Bcl11b) is a transcriptional regulator (zinc finger protein) that functions by direct, sequence-specific DNA binding activity or by recruitment to the promoter template by interaction with COUP-TF family member [11], [12], [13], [14]. The *CTIP2* gene (both mouse and human) contains 4 exons and the majority of the CTIP2 open reading frame is encoded by exon 4. Kominami's group has reported two alternatively spliced transcript variants, which encode distinct isoforms called CTIP2 long (containing exon1, 2,3 and 4) and CTIP2 short (lacking exons 2 and 3), [15]. Actual CTIP2 is the one lacking exon 3. SK-N-MC human neuroblastoma cells and Jurkat cells are known to endogenously express two splice variants of CTIP2 with a molecular weight of 95.5 and 88.5 kDa, respectively [14], [16]. Bcl11b has been suggested to function as tumor suppressor in cells, primarily based on human loss of homozygosity (LOH) studies. Several reports have described a link between chromosomal rearrangements of CTIP2 and human T cell acute lymphoblastic leukemia (T-ALL) [17]. An association between p53 and CTIP2 in mice has been implicated in the development of thymic lymphomas [15] and the amplification and/or translocation of CTIP2 is thought to play a role in human leukemogenesis [18]. Recently, it has been shown that CTIP2 is expressed early during mouse development as well as in the adult animal, and in both cases, expression is most predominant in the skin/epithelial structures, CNS and thymus [19], [20]. Mice lacking CTIP2 die at birth and exhibit epidermal differentiation defects and a compromised epidermal permeability barrier [21]. Moreover, CTIP2 expression in adult wild type mouse skin was observed to increase dramatically during wound healing, and in pathological conditions particularly in the proliferating cells of the wound bed (Ganguli-Indra et al., unpublished work). Taken together, these results demonstrate that CTIP2 could be of potential importance in human health and highlight the need for additional information regarding their function and expression patterns in pathological diseases and cancer. Since CTIP2 was upregulated in our wound healing experiments and as wound healing and cancer have key similarities where cancer is defined as “*wound that never heals*” [22], we were interested to look at CTIP2 expression in HNSCC. The main aim of this work is to understand the cellular and molecular basis of CTIP2 overexpression in HNSCC and to establish it as a novel biomarker in human HNSCC. In this study, we have determined CTIP2 expression in several human normal, and HNSCC cell lines. We also evaluated CTIP2 expression pattern by immunohistochemistry (IHC) and qRT-PCR, and determined correlation between clinicopathological factors and CTIP2 expression in a panel of human HNSCC samples.

## Materials and Methods

### Tissue samples

Paraffin embedded tissue sections from 40 different Head and neck tumors for immunohistochemistry, cDNA samples from 28 head and neck tumor and 10 normal uvula cDNA samples for qRT-PCR (normal uvulas were obtained from patients surgically treated for head and neck cancer and were at least 2 cm. away from the tumor lesion) were obtained following Institutional review board (“Comité de recherche clinique du Centre Paul Strauss.” (Président: Pr Patrick DUFOUR) approval. Informed written consents were obtained from patients undergoing surgery as a primary treatment, without previous radiation or chemotherapy. The tumors were classified according to TNM stage (tumor, node, metastasis) based on the UICC criteria (*Sobin LH, Wittekind C, 1997, TNM classification of malignant tumors. New York. Willey-Liss, Inc.*). Histopathological differentiation status was defined depending on the degree of keratin pearl formation, keratinization, overall resemblance to normal squamous epithelium, according to WHO criteria [*WHO. Histological typing of upper respiratory tract tumors. K.Shanmugaratnam and LH Sobin (eds.). International Histological Classification of Tumors. 1978, Vol. 19. Springer-Verlag (Geneva)*]. Normal samples were collected from the farthest margin of the surgical resections (uvula). All the above mentioned samples were obtained from our collaborator Joseph Abecassis, Centre Paul Strauss, Strasbourg, France.

### Histopathological features of HNSCC

**Legends for:**

**Differentiation status**

1: well-differentiated
2: moderately differentiated
3: poorly differentiated (Poorly differentiated tumors are the less differentiated squamous cell carcinomas).
4: undifferentiated (correspond to epithelial neoplasms without evidence of squamous or glandular differentiation)

**T size**

1 = T1, tumor 2 cm. or less in greatest dimension
2 = T2, tumor 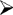
2 cm. and 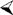
4 cm.
3 = T3, tumor 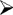
4 cm
4 = T4, tumor invades adjacent structures

**Tumor Staging**

Stage I : T1N1M0
Stage II: T2N0M0
Stage III : T3N0M0,
 T1,T2,T3,N1M0
Stage IV : T4N0,N1M0
 Any T, N2, N3, M0
 Any T, any N, M

#### Immunohistochemistry

Formalin-fixed and paraffin-embedded HNSCC sections were used in our studies. The sections were deparaffinized in xylene, dehydrated through graded alcohols, and placed in 0.1% hydrogen peroxide to quench any endogenous peroxidase activity. A 5 minute, 750 W microwave pretreatment in citrate buffer (pH 6.0) was repeated 4 times and followed by treatment with 10% normal rabbit serum for 30 minutes. to block nonspecific antibody binding. The slides were then incubated with a rat CTIP2 monoclonal antibody, 1/300 dilution (Abcam product number 18465; clone 25B6) in a humid chamber at 4°C overnight. Secondary antibody staining was carried out with a biotin-labeled rabbit anti-rat antibody 1/500 dilution (Jackson Immuno Research Laboratories, INC, catalogue number: 112-065-143) for 2 hrs. at 37°C, followed by incubation with a streptavidin-biotin horseradish peroxidase complex (Vector Laboratories, catalog number: SA-5704). Detection was done using DAB+ substrate (Vector: peroxide substrate kit, SK-4100) for 10 minutes. Counterstaining them with Mayer hematoxylin before dehydration and mounting. We analyzed the expression of CTIP2 by comparing the staining intensities between the different samples with and without addition of primary antibody. The staining intensities which were compared were done in the same time and under same conditions. We performed the staining experiment several times and the results were consistent. The analyses of CTIP2 expression were independently performed by two investigators. Staining intensities for each section were as followed: 0, no staining; 1 weak staining; 2 moderate staining; and 3 strong staining. A tumor was considered CTIP2-positive if more than 10% of the tumor cells demonstrated positive nuclear staining.

For immunofluorescence co-staining studies, paraffin sections were rehydrated as described above and processed as described [19]. Slides were then incubated overnight in a humidified chamber with CTIP2 and BMI1 (Abcam: ab14389, 1/100), cytokeratin 10 (Abcam: ab9026, 1/100, ) and Ki-67 (Abcam: ab15580, 1/200) primary antibodies. Primary antibody incubation was followed by three washes with PBST and incubation with fluorescently-labeled [Cy2 (1∶250) or Cy3 (1∶500) (Jackson ImmunoResearch)] secondary antibody for 2 hours. Nuclei were counterstained with DAPI. Finally, sections were rinsed with PBST, dehydrated through sequential washes in 50%, 70%, 95%, and 100% ethanol and then cleared in xylene. Slides were mounted with DPX mounting media and allowed to dry overnight. Images were captured at 40× magnification using Leica DMRA fluorescent microscope and Hamamatsu C4742-95 digital camera and processed using OpenLab software and Adobe Photoshop 7.0.

#### Cell culture and Western blotting

Cells were freshly thawed, cultured in their respective medium using the 3T3 protocol. When the cells reached 75 to 80% confluency we change the medium and the next day the cells were harvested and lysed in Laemmli buffer containing a protease inhibitor cocktail (Complete; Roche) and 5 mM Dithiothreitol and boiled. The whole-cell extracts (20 µg protein) were subjected to SDS-PAGE and electro-blotted to nitrocellulose membranes. The membranes were blocked in 5% non-fat dry milk in 10 mM Tris–HCl, pH 7.8, 150 mM NaCl, 0.1% Tween 20, and incubated overnight with the rat monoclonal 25B6 to CTIP2 diluted 1/1000 in the blocking buffer and developed using Super Signal West Pico Chemiluminescent Substrate (Thermo scientific Pierce).

### Cell lines used in this study

- GM 193: lymphoblast, normal EBV transformed (NIGMS Human Genetic Mutant Cell Repository)
- HUVEC: umbilical cord, endothelium, normal (ATCC CRL-1730)
- GM 3348: skin, normal fibroblasts (NIGMS)
- HS677Tg Fibroblasts, tongue, normal (ATCC CRL-7408)
- Hek-a: Primary human epidermal keratinocytes from adult skin (HEKa, SKU# C-005-5C, Cascade)
- Hacat: Spontaneously immortalized non-tumorigenic human skin keratinocytes (IGBMC cell culture service, DKFZ)
- SCC4: human squamous cell carcinoma, tongue (ATCC CRL-1624)
- Cal27: squamous cell carcinoma, tongue (IGBMC cell culture service, Dr Merlin)
- SCC25: human squamous cell carcinoma, tongue (ATCC CRL-1628)
- SCC15: human squamous cell carcinoma, tongue (ATCC CRL-1623)
- SCC9: human squamous cell carcinoma, tongue (ATCC CRL-1629)
- CAL33: Tongue cancer, elevated EGFR, contact inhibited tongue cancer (IGBMC cell culture service, Dr Merlin)
- KB: Epidermoid carcinoma, mouth (IGBMC cell culture service, Dr Merlin)
- HSC2: human squamous cell carcinoma, oral (IGBMC cell culture service)
- A253: epidermoid carcinoma, submaxillary salivary gland, (ATCC HTB-41)
- HAN HN2: head and neck tumor (IGBMC cell culture service).
- RPMI 2650: human squamous cell carcinoma, nasal septum, quasi-diploid (ATCC CCL-30)
- Det 562: human epithelial carcinoma, pharynx, metastatic (ATCC CRL-7919)
- Fadu: human squamous cell carcinoma, pharynx, primary, tumorigenic (IGBMC cell culture service, Dr Merlin)
- Hep 2: epidermoid carcinoma, larynx (ATCC CCL-23)
- MCF7: human caucasian breast adenocarcinoma (ATCC HTB-22)
- MCF10A: epithelial cell line, mammary gland, non-tumorigenic. (ATCC CRL-10317)

#### Quantitative real time PCR (QRT-PCR)

Reverse transcription was performed as described [23]. Briefly, reverse transcription was performed using 1 µg total RNA, random primers, and the superscript II RT-PCR system (Life Technologies). qPCR was performed on 1/50 dilutions of the CDNA samples using SYBR Green Supermix (qiagen) in a Applied Biosystems 7500 Real-Time PCR system as described [24]. All reactions were performed in triplicate. Melting curve analyses were performed to ensure the specificity of q-PCR. Primer sets used to test the expression of the two forms of CTIP2 were designed using Primer3 Software. Primers used for mouse *mBmi1* were as described [25]. Data analysis was performed using the 2^−ΔΔ*C*^_t_ method described previously [26]. Ribosomal phospho-protein PO (*RPLPO*) and *HPRT* was used as reference gene. qRT-PCR-based gene expression values between the groups were compared by *t test* or *1 way* ANOVA.

### Primers used for Real Time qRT-PCR

*CTIP2_L_*(hCTIP2 E2-E3):(F) 5′-ATCTGTCCCAAGCAGGAGAA-3′, (R) 5′-GTCTGACCCTCACCCTGAGT-3′; *CTIP2*(hCTIP2 E2-E4):(F)5′-AGCAGGAGAACATTGCAGGTA-3′, (R)5′-GGAAATTCATGAGCGGGGACT-3′.

#### Statistical Analysis

The means and standard errors of qRT-PCR data among different samples were calculated, and the differences between them were determined by either *t test* or 1 way ANOVA, where p<0.05 is regarded as significant.

## Results

### 

#### Expression pattern of CTIP2 in human HNSCC

**Figure 1:**
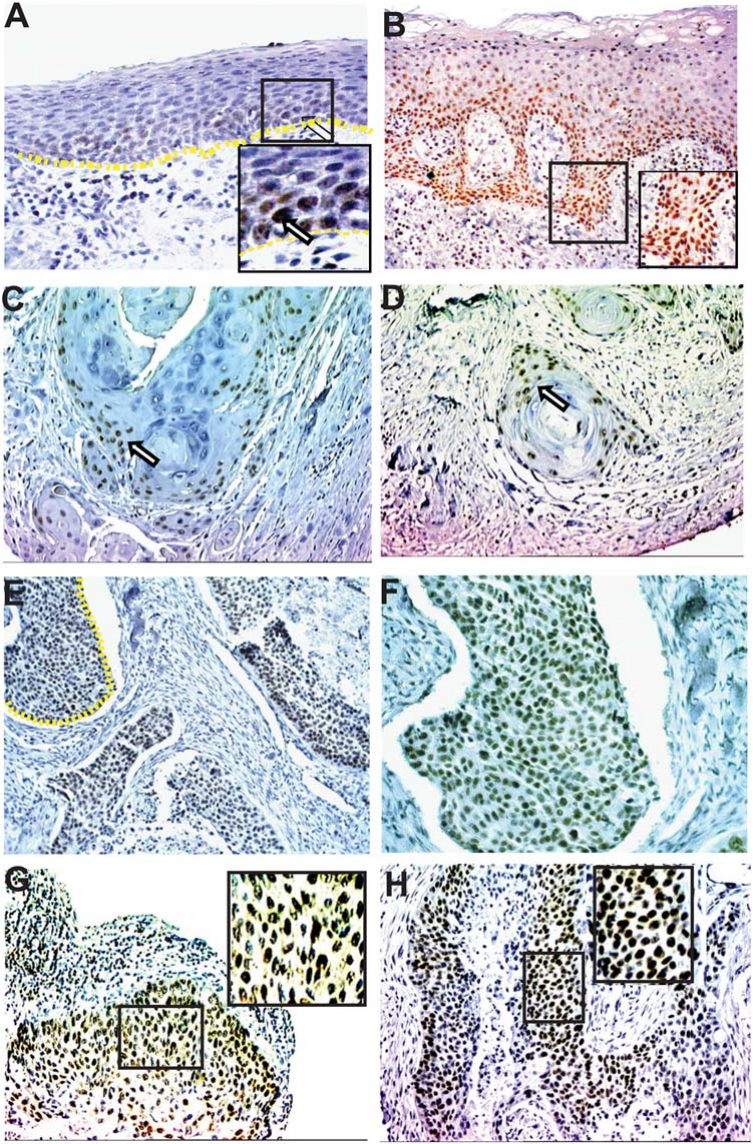
Immunohistochemical analysis of CTIP2 in HNSCC. (A) Expression in adjacent Normal epithelium is restricted to basal cell layers. (B) Expression in dysplasia is stronger than normal and extended to differentiated cell layers. (C–D) In well differentiated tumors, positive staining is restricted to the basal layers at the periphery of the keratinized horn pearl. (E–F) Homogeneous expression of CTIP2 was observed in tumor cells in moderately differentiated tumors. (G–H) Expression of stronger intensities (score 3) of CTIP2 was observed in poorly differentiated tumors. Arrows indicates the CTIP2 positive staining. Yellow dotted lines represents the margin of epidermis and dermis (A), and margin of undifferentiated tumor cell clusters (E) which is magnified in (F). The pictures in the insets (A, B, G and H) are magnified. (Original Magnification: 20×).

**Figure 2:**
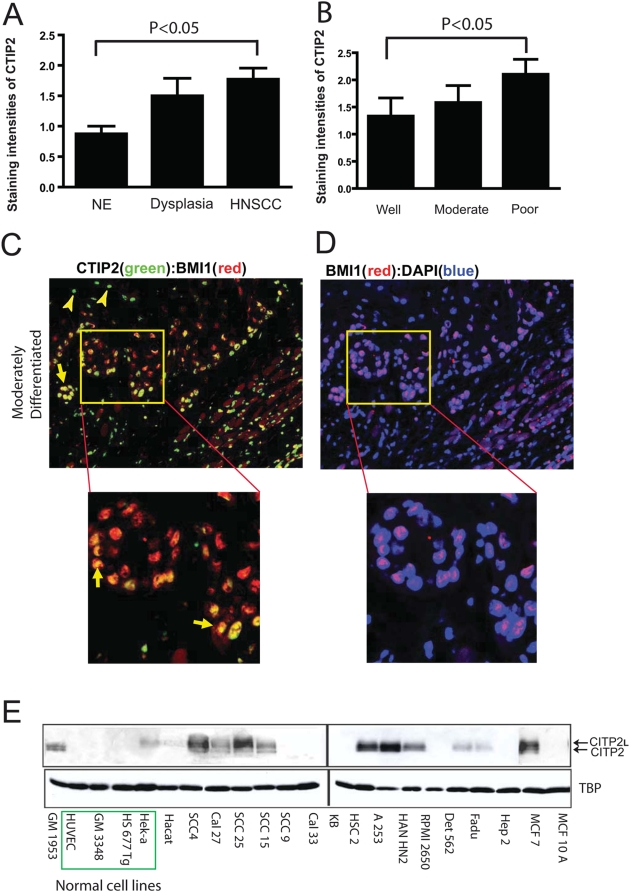
Staining intensities of CTIP2 by immuno-histochemistry in HNSCC. Staining intensities were 0–3 as described in materials and method sections. (A) Staining intensities of CTIP2 in normal epithelium (NE), dysplasia and HNSCC. Plotted are mean measurements (±S.E.M). *p<0.05. (B) Staining intensities in different histological grades of HNSCC. Plotted are mean measurements (±S.E.M). *p<0.05. (C–D) Co-staining of CTIP2 and BMI1 protein. The insets are magnified. Yellow arrows show the CTIP2 and Bmi1 double positive cells. Yellow arrowhead indicates single positive cells. (E) CTIP2 expression in HNSCC cell lines by western blot analysis. CTIP2 antibody recognizes the two isoforms (Long and the actual CTIP2). Green box represents the normal cell lines.

In order to localize CTIP2 expression in HNSCC, we performed immunohistochemistry on paraffin embedded sections of Head & Neck tumors (n = 40) and normal samples (n = 12) using high heat antigen retrieval technique and the previously described CTIP2 antibody [19], [21]. Positive staining for CTIP2 was found to be exclusively nuclear. Negative control on sections stained without primary antibody or on skin sections from CTIP2 null mice confirmed no staining [21]. In tumor adjacent normal epithelium CTIP2 was expressed exclusively by the keratinocytes of the basal layer and no expression was detected in cells of the spinous cell layer or granular layer. (Arrows in Fig. 1A, see inset). In dysplasia samples, CTIP2 expression was stronger compared to the normal epithelium and also the expression was extended to the spinous cell layers (Fig. 1B and *inset*). In well differentiated tumors, CTIP2 staining was observed in the outer layer of the keratinized horn-pearls clusters (Fig. 1C and D). Stronger and homogeneous expression of CTIP2 was observed in moderately and/or poorly differentiated tumors (Figs. 1E–H) compared to the well differentiated tumors. We compared the intensities of CTIP2 staining (0–3) (described in materials and methods) between the adjacent normal, dysplasia and different histological grades of Head & Neck tumor (Fig. 2A and 2B). Out of the 40 different samples we analyzed, 12 of them had adjacent normal epithelium and 4 of them had dysplasia. Compared to normal epithelium, CTIP2 expression was strongly and significantly up-regulated in HNSCC samples (P<0.05) (Fig. 2A). The strongest intensity of CTIP2 staining (level 3) was observed in 11% well differentiated tumors, 25% of the moderately and in 58% of the poorly differentiated tumors compared to no staining of this intensities in adjacent normal epithelium (Fig. 1, Fig. 2A & B and Table 1). Overall, we observed an increase in the intensity of CTIP2 expression with the progression of carcinoma in all our samples analyzed. Thus, our IHC results demonstrates that CTIP2 expression is elevated in HNSCC, and that CTIP2 expression is linked to poor differentiation state of the tumor.

**Table 1:** Staining intensities and percent positivity in normal epithelium and in HNSCC.

A					
		Staining Intensities			
	n*	0	1	2	3
**Adjacent Normal**	12	2	10	0	0
**Dysplasia**	4	0	1	2	1
**HNSCC-Well**	9	2(23)	3(33)	4(44)	0(0)
**HNSCC-Moderately**	12	2(17)	4(33)	3(25)	3(25)
**HNSCC-Poor**	19	2(10)	3(16)	3(16)	11(58)

B					
Tumor differentiation		Percent positivity			
		0–25	25–50	50–75	100
**Adjacent Normal**	12	12	0	0	0
**Well**	9	6	3	0	0
**Moderately**	12	4	6	2	0
**Poor**	19	5	6	8	0
**Tumor Stage**					
**Stage I–II**	22	14	6	2	
**Stage III–IV**	18	5	6	7	0

In brackets are the percentages of specific tumor types.

Several reports have shown that polycomb group transcription factor BMI1, is essential for the self renewal of hematopoietic, neuronal stem cells as well as cancer stem/progenitor cells [27], [28], [29]. We hypothesized that CTIP2 might label and modulate self renewal of tumor initiating/cancer stem cells in HNSCC. We therefore performed immunoflourescent co-staining for CTIP2 and BMI1 protein, in well, moderately and poorly differentiated HNSCC samples to see if some of the CTIP2 expressing cells also express the cancer stem cell (CSC) marker BMI1, previously implicated in self renewal and tumorigenesis [30], [31], [32], [33], [34], [35], [36], [37]. Staining for BMI1 was observed mostly in the basal cell layer and decreased towards the outer cell layers. In well and moderate to poorly differentiated tumors, majority of the BMI1 expressing cells showed strong nuclear co-localization of CTIP2 and BMI1 proteins (CTIP2+/BMI1+) (Fig. 2C & D). Besides, some of the cancer cells were stained single positive for CTIP2 (CTIP2+/BMI1^−^) (Fig. 2C, *inset*). Interestingly, qRT-PCR performed on the CTIP2 mutant mice skin, using mouse specific *Bmi1* primers, showed significant down regulation of *Bmi1* expression, suggesting a possible regulation of *Bmi1* gene by Ctip2 (Fig. S1) [21]. Furthermore, promoter analyses of mouse and human *BMI1* gene revealed presence of several putative CTIP2 binding sites in the proximal and distal promoter region (data not shown) [12], [14]. Altogether, our results suggest existence of distinct subsets of cancer cells which express CTIP2 and underscore the use of CTIP2 and BMI1 co-labeling to identify tumor initiating cancer stem cells (CSC).

#### Expression of CTIP2 in human cell lines

CTIP2 protein expression was detected by western blotting in a panel of cell lines which included normal, HNSCC and other types of carcinomas (see material and methods) (Fig. 2E). We detected weak expressions of CTIP2 in primary human epidermal keratinocytes (Hek-a) and in spontaneously immortalized non-tumorigenic human skin keratinocytes (Hacat) [Fig. 2E]. CTIP2 expression was undetectable in some cell lines from umbilical cord endothelium (HUVEC), skin and tongue fibroblast (GM3348 & HS677Tg). In contrast, very high level of CTIP2 was detected in head and neck cell lines (SCC4, SCC25, HSC2, HAN HN2) and also in other carcinomas such as epidermoid carcinoma from submaxillary salivary gland (A253) and breast carcinoma (MCF7) (see Fig. 2E). Surprisingly, weaker expression was detected in few Head and neck cell lines such as Det562 and Fadu, and no expression at all in RPMI 2650. In most of the cases, CTIP2 expression level was lower than our internal control TATA box binding protein (TBP). Altogether, these results demonstrate that most of the HNSCC cell lines have increased expression of CTIP2.

#### Co-labeling of CTIP2 with a proliferation & differentiation marker

**Figure 3:**
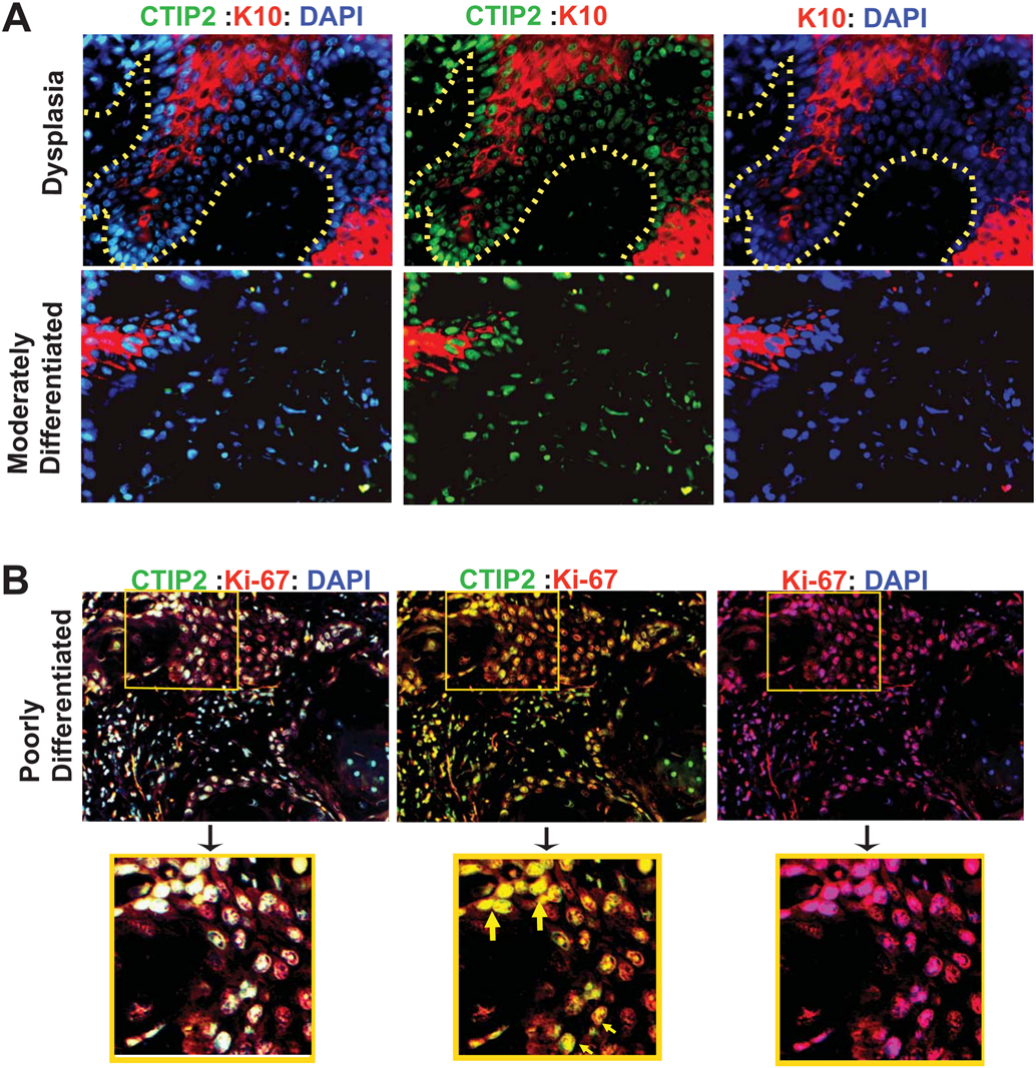
Co-staining of CTIP2, cytokeratin 10 and Ki-67 using immunofluorescence. (A) Co-staining of CTIP2 and differentiation marker cytokeratin 10 in dysplasia and in moderately differentiated tumor. CTIP2 in green, Cytokeratin 10 in red and DAPI in blue. Yellow dotted lines represents the margin of epidermis and dermis. (B) Co-staining of CTIP2 and a proliferation marker Ki-67 in poorly differentiated tumor. Insets are in yellow boxes and are magnified. Yellow arrows shows the CTIP2-Ki-67 double positive cells. (Original Magnification: 20×).

To confirm if CTIP2 is linked to the proliferation and differentiation of HNSCC, we used immunofluorescence method to co-label CTIP2 with a proliferation (Ki-67) and a differentiation [cytokeratin 10 (K10)] marker. We observed cytoplasmic expression of K10 either in clusters or in scattered areas of the dysplasia samples and also in tumors with well or moderately differentiated status (Fig. 3A). In dysplasia samples, most of the supra basal layer cells which were positive for CTIP2 also expressed K10. In well to moderately differentiated tumor samples with low level of CTIP2 expression, Cytokeratin 10 was expressed in clusters (Fig. 3A). Some of the CTIP2 positive cells also expressed K10, and some CTIP2 positive cells were negative for K10. In poorly differentiated tumors with less differentiation (less K10 positivity), high levels of CTIP2 was observed which did not express K10. We have recently shown that CTIP2 colabel with proliferation marker Ki-67 in normal human skin and in eczematous skin [38]. In the same line, in our co labeling experiment in HNSCC, most of the CTIP2 positive cells were also found to be co-labeled with Ki-67 (Fig. 3B, see double positive yellow cells). Altogether, our results with K10 and Ki-67 shows that CTIP2 expression is linked to tumor differentiation state and proliferation. Poorly differentiated tumors which are less differentiated contain more proliferative cells, and hence have higher CTIP2 expression. These results on cytokeratin 10 and Ki-67 staining pattern confirms that CTIP2 is linked to differentiation status of the tumor. Less differentiated tumor (poorly differentiated tumors) express high CTIP2 levels with proliferative nature (colabel with Ki-67) and express very low levels of cytokeratin K10.

#### Expression of different CTIP2 transcript in human HNSCC

**Figure 4:**
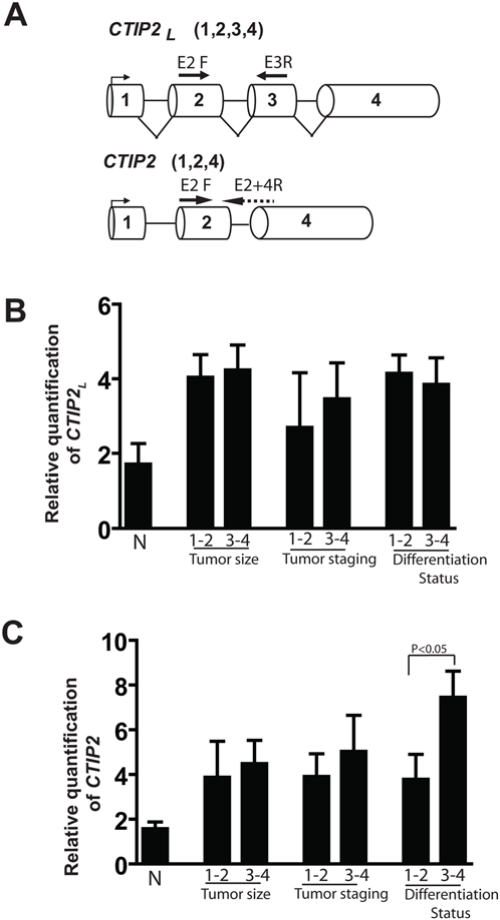
Scheme of *CTIP2* transcripts and the expression pattern of *CTIP2_L_* and *CTIP2* in HNSCC. (A) Two transcripts of human *CTIP2* are shown. *CTIP2* with all the 4 exons, is the long form of *CTIP2* (*CTIP2* *_L_*), and, lacking exon 3 is the actual *CTIP2*. Primers for the long form were taken from Exon 2 for Forward and exon 3 for reverse, and for the shorter form, the forward from boundary of Exon 2 & 4 and the reverse from exon 4. Expression of the *CTIP2* long (B) and *CTIP2* (C), by qRT-PCR in HNSCC (*n = 28*) and 10 normal uvulas based on T size, Tumor staging, and differentiation status. Plotted are the Relative quantification levels normalized with *RPLPO* as S.E.M±. p values were not significant between the groups in (B) but a significant correlation of higher *CTIP2* expression was observed with a poorer histological grade of the tumor, and a trend was noted in a relationship between expression and advanced T or clinical stage in (C) [P<0.05].

Since the CTIP2 antibody we used in our earlier experiments could not distinguish between the specific CTIP2 isoforms by IHC, we were interested to learn which isoform(s) of CTIP2 were relevant to HNSCC disease progression by quantitative real time PCR (qRT-PCR). Human *CTIP2* has three alternatively spliced transcript variants, which encode distinct isoforms (see Fig. 3A). In our present study, we have referred *CTIP2* with all the 4 exons as *CTIP2* long (*CTIP2* ***_L_***), the variant lacking exon3 which is the actual *CTIP2* as *CTIP2* (Fig. 3A), and the variant which lacks both Exon2 and 3 as *CTIP2* short (not shown). qRT-PCR was performed for *CTIP2* ***_L_*** and *CTIP2*, on RNA extracted from 28 different HNSCC samples and amplifications were normalized using *RPLPO* as an internal control. We also included 10 normal uvulas to compare the expression levels of the transcripts along with HNSCC samples. To distinguish between the two forms, we have designed specific primers from exon 2 and exon 3 for *CTIP2* ***_L_***, and exon 2 and overlapping exon 2 and 4 for *CTIP2* (see Fig. 3A). The samples from different tumor size (T size), Tumor staging (T staging) and differentiation status (see material methods for details) were divided into early cancer (1–2) and late cancer (2–4), respectively. A significant number of samples with normal to high mRNA expression was observed among patients with T1-T4 tumor size and with well, moderate to poor differentiation status, and regional metastasis. However, statistical studies showed no statistical significance of any correlation between mRNA expression and any of the clinicopathological parameters with the *CTIP2* ***_L_*** (see Fig. 4A and Table 2). On the contrary, there was a significant correlation of higher *CTIP2* expression with a poorer histological grade of the tumor, and a trend was noted in a relationship between expression and advanced tumor (T size) or Tumor staging (Fig. 4B and Table 2) (P<0.05). Altogether, these results suggest that although both the forms of *CTIP2* are elevated in HNSCC only the actual *CTIP2* is linked to the poor differentiation status of the tumor.

**Table 2:** Correlation between *CTIP2* mRNA expression levels and Clinicopathological characteristics in HNSCC.

		Total		
	Total		*CTIP2_L_*	*CTIP2*
All cases	28		P value	P value
**Tumor differentiation status**		***t test***	0.734	0.0439
Well-Moderately	18			
Poor-Undifferentiated	10			
**Tumor location**		***1 way anova***	0.0716	0.2476
Hypopharynx	8			
Oral cavity	10			
oropharynx	6			
larynx	4			
**Tumor size**		***t test***	0.8394	0.5511
T1–T2	19			
T3–T4	9			
**N Stage**		***t test***	0.6697	0.9862
N0	13			
N+	15			
**Tumor Staging**		***t test***	0.6737	0.3217
I–II	9			
III–IV	19			

Statistical analysis was performed as described in material and methods sections.

## Discussion

In the present study, we demonstrated for the first time over-expression of transcriptional regulatory protein CTIP2 in head and neck squamous cell carcinomas using IHC and qRT-PCR analysis. CTIP2 overexpression was detected in different human HNSCC cell lines as well as in HNSCC tumor specimens of different grades and/or differentiation stages. The protein was detected in several HNSCC cell lines, besides a breast adenocarcinoma cell line (MCF7), and its expression was relatively low compared to an internal control. Highest level of expression was observed in HSC2 (oral) and A253 (salivary gland) cells, and the lowest in SCC9 and Cal 33 (tongue), KB (mouth), Hep2 (larynx) and RPMI 2650 (nasal septum). The variation in the level of expression among different lines may depend on factors such as tumor stage and/or differentiation status of the tumor cell lines.

In tumor sections, CTIP2 protein expression was associated with histological changes such as proliferation and differentiation, and its increased expression was linked to poorer differentiation status of those tumors. We were able to detect expression of CTIP2 protein in normal epithelium and in tumor cells with HNSCC characteristics within the same tissue sections. Intensities of staining increased from well, moderately to poorly differentiated tumors. Dramatic increase in CTIP2 expression was found in dysplasia where expression was observed all the way up to the differentiating cell layers, compared to the normal where expression was restricted to the basal cell layer. We also determined the expression pattern of the two different isoforms of CTIP2 [CTIP2_L_ and CTIP2] in progression of HNSCC, using qRT-PCR analyses and by designing primer specific for each isoform. Although *CTIP2_L_* was elevated in HNSCC, the actual *CTIP2* (*CTIP2*) was found to be more relevant to the disease progression. The *CTIP2* mRNA level correlated well with the protein expression level obtained by IHC and western.

It was recently demonstrated that human HNSCC contain tumorigenic cancer stem cells (CSC) with an ability to self renew and differentiate [36], [39]. In the same line, co-labeling experiments with CTIP2 and CSC marker BMI1 highlighted the existence of a distinct subset of cancer cells (BMI1+/CTIP2+) with possible stem cell characteristics. It has been already shown that CTIP2 is widely expressed in normal mouse skin [19]. Some, but not all, of the cells present within hair follicle bulge were also found to co-express CTIP2, keratin K15, but not CD34, indicating that a subset of K15+ CD34− skin stem cells may express CTIP2 [19]. It is possible that CTIP2 might regulate a subset of epithelial stem cell population in skin as well as in HNSCC. Recent contradictory reports on widespread [34] versus restricted [37] expression pattern of CSC marker CD44 in HNSCC has opened up discussion to identify novel CSC markers in this cancer type. The present study underscores the possible use of CTIP2 and BMI1 co-labeling to identify cancer stem cells (CSC) in HNSCC and suggests a transcriptional regulation of Bmi1 gene by CTIP2. The mechanisms of differential expression of CTIP2 and regulation of gene expression by CTIP2 in HNSCC remain to be elucidated.

CTIP2 protein is already known for its involvement in T-cell lymphomas and implicated in mouse and human leukemias [40]. CTIP2 is also known to make the DNA more vulnerable to replication stress and damages, thus being involved in maintenance of genomic stability [41]. Mice with a germ line deletion of the CTIP2 die after birth and exhibit defects in epidermal proliferation and terminal differentiation [21]. CTIP2 is also expressed in human skin and is upregulated in skin from atopic dermatitis patients [38]. Since CTIP2 has a role in maintenance of skin homeostasis, it could play a significant role in regulating proliferation and differentiation of the tumor cells in HNSCC. In the present study we found lower CTIP2 expression in more differentiated tumor type and CTIP2 expression increased with loss of differentiation. Moreover, most of the Ki-67 positive cells also were positive for CTIP2, indicating proliferative potential of CTIP2 positive cells. However, the underlying molecular mechanisms and role of this protein in epithelial carcinogenesis is yet unknown. Mouse models of human cancer play an important role in understanding the mechanisms of carcinogenesis and have accelerated the search for finding new molecular targets for cancer therapy [42]. Therefore, generating transgenic mouse model of CTIP2 overexpression using epithelial specific promoter would be a useful tool to study the role of CTIP2 in understanding the molecular mechanisms of HNSCC development and progression.

## Supporting Information

Figure S1 Expression of mouse Bmi1 in Ctip2 mutant embryonic skin by qRT-PCR. Reduced expression of Bmi1 was observed in the Ctip2 mutant mice. Plotted are the Relative quantification levels normalized with HPRT as mean±S.E.M. p values between wild type and Ctip2 mutant were significant (p<0.05).
(0.60 MB EPS)
