# Correction: CTIP2 Expression in Human Head and Neck Squamous Cell Carcinoma Is Linked to Poorly Differentiated Tumor Status

**DOI:** 10.1371/annotation/b01c646f-14c7-4aac-8812-9ed4f847c857

**Published:** 2009-04-30

**Authors:** Gitali Ganguli-Indra, Christine Wasylyk, Xiaobo Liang, Regine Millon, Mark Leid, Bohdan Wasylyk, Joseph Abecassis, Arup K. Indra

The middle initial of the last author's name was omitted. The correct name is: Arup K. Indra. 

